# Vascular Tissue Engineering: Effects of Integrating Collagen into a PCL Based Nanofiber Material

**DOI:** 10.1155/2017/9616939

**Published:** 2017-08-28

**Authors:** Ulf Bertram, Dominik Steiner, Benjamin Poppitz, Dirk Dippold, Katrin Köhn, Justus P. Beier, Rainer Detsch, Aldo R. Boccaccini, Dirk W. Schubert, Raymund E. Horch, Andreas Arkudas

**Affiliations:** ^1^Department of Plastic and Hand Surgery, University Hospital of Erlangen, Friedrich-Alexander-University of Erlangen-Nürnberg (FAU), Erlangen, Germany; ^2^Institute of Polymer Materials, Friedrich-Alexander-University of Erlangen-Nürnberg (FAU), Erlangen, Germany; ^3^Department of Materials Science and Engineering, Institute of Biomaterials, Friedrich-Alexander-University of Erlangen-Nürnberg (FAU), Erlangen, Germany

## Abstract

The engineering of vascular grafts is a growing field in regenerative medicine. Although numerous attempts have been made, the current vascular grafts made of polyurethane (PU), Dacron®, or Teflon® still display unsatisfying results. Electrospinning of biopolymers and native proteins has been in the focus of research to imitate the extracellular matrix (ECM) of vessels to produce a small caliber, off-the-shelf tissue engineered vascular graft (TEVG) as a substitute for poorly performing PU, Dacron, or Teflon prostheses. Blended poly-*ε*-caprolactone (PCL)/collagen grafts have shown promising results regarding biomechanical and cell supporting features. In order to find a suitable PCL/collagen blend, we fabricated plane electrospun PCL scaffolds using various collagen type I concentrations ranging from 5% to 75%. We analyzed biocompatibility and morphological aspects* in vitro*. Our results show beneficial features of collagen I integration regarding cell viability and functionality, but also adverse effects like the loss of a confluent monolayer at high concentrations of collagen. Furthermore, electrospun PCL scaffolds containing 25% collagen I seem to be ideal for engineering vascular grafts.

## 1. Introduction

The production and integration of tissue engineered vascular grafts (TEVGs) as a surrogate for autologous veins and arteries as well as common graft materials, such as polyurethane (PU), Dacron, or Teflon, have been a focus of research for more than a decade [1]. While common graft materials perform poorly at small calibers (<5 mm) [2, 3], the current clinical standard, the autologous vascular graft, is associated with donor-site morbidity and limited availability [4], especially for the growing number of patients suffering from multiple morbidities, including chronic vascular diseases. US surgeons perform cardiovascular bypass surgery on some 700.000 patients every year [5].

Vascularization of an engineered tissue after implantation poses a decisive challenge of tissue engineering (TE) in general [6]. As TE targets the improvement, maintenance, or restoration of organs [7], the size of the products produced tends to exceed the diffusional limit for oxygen and nutrients [8]. Often enough the engineering of thick tissues in particular makes the integration of a vascular graft mandatory [9].

There have been numerous attempts to produce a suitable vascular substitute using different techniques, such as decellularized xenografts [10], fabrication of a permanent synthetic or biodegradable scaffold, or “sheet-based tissue engineering” entirely without the use of a scaffold [11]. The common idea is to gain or imitate an extracellular matrix (ECM) [12] that allows cells, such as endothelial cells (ECs) and smooth muscle cells (SMCs), to attach to the material and provide adequate biomechanical durability [13].

Electrospinning is a widespread method for the production of vascular graft material [4, 14, 15]. It is a commonly used method to produce nanofiber based, 3-dimensional scaffolds from different polymers or polymer blends. The process itself allows controlling fiber diameter and porosity of the desired graft material [16].

Various biodegradable polymers such as poly(lactic-co-glycolic acid) (PLGA), polylactide (PLA), or poly(glycerol sebacate) (PGS) have shown promising results* in vitro* but did not meet the required biomechanical features* in vivo* [4, 17]. Poly-*ε*-caprolactone (PCL) has been shown to have excellent biomechanical features, a slow degradation rate and most importantly very good biocompatibility [18]. As an important structural element of native vessels, collagen is also used to imitate the ECM component of electrospun nanofiber grafts [4, 13, 15].

Collagen allows ECs and SMCs to attach to biopolymers and is a frequently used matrix in TE products and regenerative medicine in general [19]. Collagen nanofibers have excellent biophysical features that make them a crucial element in TE. They transmit forces, prevent premature mechanical failure, provide biological signals to adherent cells, and promote tissue regeneration [20]. At least 20 different types of collagen have been identified [19]; the most prevailing of these are types I, II, and III [20].

One of the most essential problems concerning an artificial vascular graft is thrombogenicity, especially at small caliber and low-flow conditions [21]. The lack of endothelialization of the graft material appears to be the decisive factor; thus the implementation of a vascular graft that comes along with a confluent monolayer of ECs has been considered as a solution for this particular problem [22, 23].

While gaining autologous differentiated ECs is associated with a considerable donor-site morbidity, endothelial progenitor cells (EPCs) can be obtained from peripheral blood of a patient relatively easy [24]. EPCs have a great angiogenic potential [25], thus making them ideal for the implementation in TEVGs.

Blending collagen with other polymers is a common practice for the fabrication of nanofiber scaffolds in order to imitate the native vessels ECM.

In this study, we focused on the collagen ratio using PCL as the basic biopolymer. We fabricated plane nanofiber scaffolds with a 5%, 25%, 50%, and 75 wt% rate of collagen type I to compare them to pure PCL scaffolds under* in vitro* conditions. We used T17b murine embryonic endothelial progenitor cells (eEPCs) and analyzed their behavior and morphology on blended nanofiber scaffolds.

## 2. Materials and Methods

### 2.1. Scaffold Production

For the fabrication of electrospun nanofiber scaffolds, pure PCL (*M*_*w*_ ~ 14.000 g/mol) (Sigma Aldrich, Schnelldorf, Germany) and different polymer blends of PCL and purified collagen type I from bovine skin (Symatese, Chaponost, France) were used to achieve collagen concentrations of 5%, 25%, 50%, and 75% (w/w). PCL and PCL/collagen (PCL/Coll) blends were then solved in 1,1,1,3,3,3-hexafluoroisopropanol (HFIP) (Sigma Aldrich, Schnelldorf, Germany) overnight resulting in a polymer ratio of 3,6% (w/v).

The general principle of electrospinning polymer nanofiber scaffolds has been described many times [12, 16]. Briefly, a glass syringe was filled with polymer solution and mounted on a syringe pump with a constant flow-rate of 2 ml/h. Additionally, 20 kV was applied to the 21-gauge stainless steel syringe needle. A grounded 6 × 9 cm aluminum (Al) plate was opposed horizontally in a needle tip-collector distance of 15 cm. The spinning procedure lasted for 2-3 h to gain a macroscopically homogenous scaffold of random pattern nanofibers. To ease the detachment of the scaffold, Al plates were placed in a 1% (w/v) sodium chloride solution overnight and dried in advance of the spinning process.

To facilitate the use of PCL and PCL/Coll scaffolds in 24-well plates, 11 mm diameter chips were die-cut from electrospun scaffolds and mounted onto Minusheet® tissue carriers (MINUCELLS and MINUTISSUE, Bad Abbach, Germany). Mounted scaffolds were then sterilized in 70% ethanol (Carl Roth, Karlsruhe, Germany) for 45 min at room temperature and dried overnight at 37°C. Sterilized scaffolds were stored in 24-well plates (Greiner Bio-One, Frickenhausen, Germany) until use.

The fiber characteristics of our scaffolds were analyzed using ImageJ™  v.1.48 (National Institutes of Health, Bethesda, MD, USA). Nanofiber diameters were measured automatically with DiameterJ, a validated plug-in for ImageJ.

### 2.2. Cell Culture

T17b eEPCs are a murine mesodermal cell line, isolated from mice embryos. They feature characteristics of EPCs and their differentiation towards ECs can be induced* in vitro* by modification of the cell culture medium [26]. Prior to cultivation of T17b eEPCs culture flasks (Greiner Bio-One, Frickenhausen, Germany) were coated with bovine skin gelatine type B (bsGel-TB) (Sigma Aldrich, Schnelldorf, Germany). Cell cultivation was performed using a HERAcell® 150i Incubator (Thermo Scientific, Waltham, MA, USA) in a humidified atmosphere (37°C; 5% CO_2_). The basal medium (BM) for T17b eEPC cell culture is based on a high glucose Dulbecco's modified Eagle's medium (DMEM) GlutaMAX® (Gibco/Life Technologies, Carlsbad, CA, USA) modified with 20% fetal calf serum (FCS) (Biochrom, Berlin, Germany), 100 U/ml penicillin (Biochrom), 100 *μ*g/ml streptomycin (Biochrom), 1 mM nonessential amino acids (NEAA) (Gibco), 2 mM HEPES buffer pH 7.5 (Gibco), and 0.1 mM 2-mercaptoethanol (Gibco). Supplementing BM with 1 *μ*M all-trans-retinoic acid (ATRA) (Sigma Aldrich) and 0.5 mM dibutyryl cyclic AMP (cAMP) (Sigma Aldrich) gained a differentiation medium (DM). T17b eEPC differentiation was induced by exposure to DM for 72 h before being seeded onto nanofiber scaffolds. Cells were detached from cell culture dishes using Accutase solution (PromoCell, Heidelberg, Germany).

### 2.3. Cell Seeding onto Nanofiber Scaffolds

To colonize the sterilized and mounted scaffolds, differentiated eEPCs were detached from cell culture dishes and counted via C-Chip (Biochrom, Berlin, Germany). 1 × 10^5^ cells were seeded on each scaffold or bsGel-TB coated 24 wells, respectively, using 50 *μ*l of DM. The cells were allowed to attach to the material for 1 h at 37°C and 5% CO_2_ before adding another 950 *μ*l of DM. For each group (bsGel-TB, PCL, PCL/Coll 5%, PCL/Coll 25%, PCL/Coll 50%, and PCL/Coll 75%) and time point (24 h, 48 h, and 72 h) 6 individual scaffolds were prepared with cells (*n* = 6). The bsGel-TB group represented the control group because bovine skin gelatine type B has been widely used as standard coating for eEPCs [26].

### 2.4. Cell Viability Assay

The detection of viable cells on nanofiber scaffolds was performed after 24 h, 48 h, and 72 h using a water-soluble tetrazolium salt (WST-8) based Cell Counting Kit 8 (CCK-8) (Sigma Aldrich, Schnelldorf, Germany). Briefly, scaffolds were washed with phosphate buffered saline (PBS) (PromoCell, Heidelberg, Germany), transferred into a new well plate, and supplied with fresh DM and ready-to-use CCK-8 solution. Each scaffold was incubated for 2 h and supernatants were analyzed photometrically at 450 nm using a Multiskan™ GO ELISA reader (Thermo Scientific, Waltham, MA, USA). For each group and time point (24 h, 48 h, and 72 h) 6 individual experiments were performed using scaffolds from one batch per group and one cell culture for each time point.

### 2.5. Cell Proliferation Assay

Cell proliferation was investigated with a colorimetric BrdU Cell Proliferation ELISA (Roche Diagnostics, Mannheim, Germany). Samples were each washed with PBS, transferred into a new well plate, and incubated with BrdU labeling solution for 24 h before evaluation. The tests were carried out following the fabricators' protocol but scaled to a well volume of 500 *μ*l. After 5 min of incubation with substrate solution, the reaction was stopped using 1 M H_2_SO_4_ and supernatants were analyzed within 5 min at 450 nm and a reference wavelength of 690 nm with an ELISA reader. Experiments were performed analogous to the viability assay.

### 2.6. DNA Fragmentation ELISA

A Cell Death Detection ELISA (Roche Diagnostics, Mannheim, Germany) was used to quantify the rate of apoptotic T17b eEPCs on the different nanofiber scaffolds. All steps were carried out corresponding to manufacturer's instructions. Briefly, cells were detached from the scaffolds or bsGel-TB 72 h after cell seeding and centrifuged at 1200 rpm for 5 min including the supernatants before lysis. After the lysis step, the supernatants containing the DNA were transferred onto an anti-histone antibody-coated microtiter plate. A second peroxidase-labeled anti-DNA antibody was added and peroxidase substrate ABTS (2,20-azino-di[3-ethylbenzothiazoline-sulfonate]) was applied to perform photometric analysis. Microtiter plates were analyzed in an ELISA reader at 405 nm and a reference wavelength of 690 nm. 6 individual scaffolds within one group, taken from the same batch, were seeded with T17b eEPCs from the same cell culture.

### 2.7. VEGF ELISA

In order to measure the amount of VEGF produced by T17b EPCs, supernatants of 6 samples of each group were gathered after 24 h, 48 h, and 72 h. Quantification of VEGF was carried out using a mouse VEGF Quantikine ELISA Kit according to company's protocol (R&D Systems, Wiesbaden, Germany). Photometric determination was accomplished by measuring the absorbance at 450 nm and reference wavelength of 540 nm by ELISA reader. Experiments were performed as mentioned above.

### 2.8. Cell Morphology Study

The entire experiment was carried out using cells from the same cell culture. For each group 3 individual scaffolds were prepared, using material from one batch.

#### 2.8.1. Sample Preparation for SEM

Nanofiber scaffolds containing T17b eEPCs were washed with PBS first and fixed in multiple steps, starting with incubation in a 0.2 M sodium cacodylate trihydrate (Sigma Aldrich, Schnelldorf, Germany) buffer, containing 0.1% glutaraldehyde (Carl Roth, Karlsruhe, Germany), 2% paraformaldehyde (Carl Roth), and 5% sucrose (Sigma Aldrich, Schnelldorf, Germany) for 1 h at room temperature. In a second step, samples were placed in a 0.2 M cacodylate buffer, containing 0.3% glutaraldehyde and 3% paraformaldehyde for 1 h, followed by a series of ethanol dilutions in rising concentration, ending at 100 vol.%.

Fixed samples were then critical point dried with EM CPD300 (Leica Microsystems, Wetzlar, Germany), removed from Minusheets, gold-palladium sputtered, and examined using an AURIGA® scanning electron microscope (SEM) (Carl Zeiss, Jena, Germany).

#### 2.8.2. Fluorescence Staining

For cytoskeleton staining, samples were fixed in a 4% paraformaldehyde solution and permeabilized using 0.3% Triton X-100 (Sigma Aldrich, Schnelldorf, Germany) in PBS. Fixed scaffolds were then incubated with rhodamine-labeled phalloidin (Molecular Probes, Eugine, OR, USA) for 1 h, followed by staining with DAPI solution (Molecular Probes) for 5 min. After staining, samples were removed from Minusheets, transferred onto glass slides, and supplied with Fluoprep (Biomérieux, Marcy-l'Étoile, France) and cover glasses. The examination was conducted using an Axio Scope.A1 reflected light fluorescence microscope (Carl Zeiss, Jena, Germany).

### 2.9. Statistical Analysis

Firstly the data was tested for Gaussian distribution. The characteristics of nanofiber scaffolds were then analyzed with Kruskal-Wallis test and Dunn's test for multiple comparisons. All assay data was analyzed using a one-way ANOVA and Tukey test for multiple comparisons. Data is either shown as box plot or depicted as mean arbitrary units ± SD. *P* values ≤ 0.05 were considered statistically significant. Statistical analysis was performed using GraphPad Prism 7 (GraphPad Software, San Diego, CA, USA).

## 3. Results

### 3.1. Scaffold Characterization

**Figure 1:**
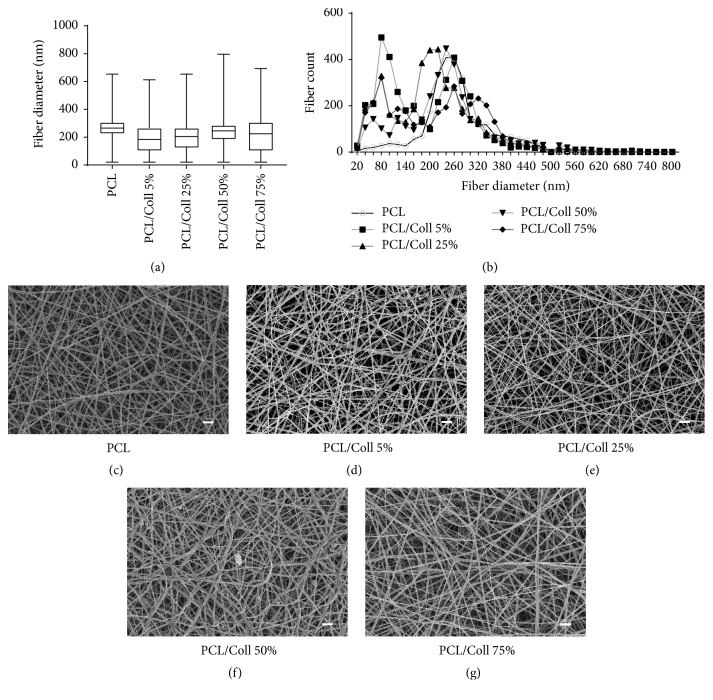
The analysis of SEM images shows that median fiber diameters of our scaffolds range from 184 nm (PCL/Coll 5%) to 265 nm (PCL) (a). Fiber diameters do not assume Gaussian distribution, as the more detailed histogram (b) depicts. (c)–(g) show SEM images of our scaffolds' surface magnified 5k-fold (scale bar *≙* 2 *μ*m).

The different scaffolds were analyzed before cell culture. The mean fiber diameters range from 191 ± 103 nm (median: 184 nm) for PCL/Coll 5% scaffolds to 274 ± 87 nm (median: 265 nm) for pure PCL scaffolds (Figure 1(a)). Furthermore, the data produced by DiameterJ indicates that the distribution of fiber diameters varies significantly between all (PCL and PCL/Coll) groups (*P* ≤ 0.05) (Figure 1(b)). Figure 1(b) also shows that there is no Gaussian distribution in fiber diameters in any group. Instead, multiple peaks of fiber diameter counts can be found in groups containing collagen. SEM images of the scaffolds display a random pattern of fiber alignment (Figures 1(c)–1(g)).

### 3.2. Effects of PCL Nanofiber Scaffolds on Cell Viability

**Figure 2:**
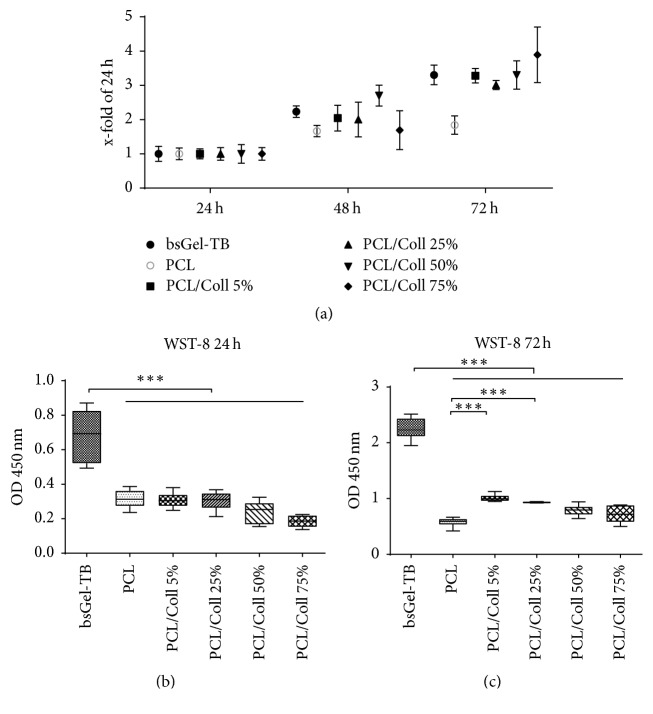
1 × 105 T17b eEPCs were seeded on bovine gelatine type B coated wells, plane PCL, or PCL/collagen I nanofiber scaffolds. Results are expressed as increase in metabolic activity in relation to 24 h (a). Absolute results suggest an overall superiority of gelatine coating after 24 h (b) and 72 h (c) compared to PCL and PCL/collagen nanofiber scaffolds regarding metabolic activity. After 72 h, PCL/Coll 5% and 25% appear to be advantageous to pure PCL scaffolds. Statistically significant differences between the different groups are indicated for ^*∗∗∗*^*P* < 0.001.

The WST-8 assay detects viable cells due to their metabolic activity. 48 hours after cell seeding all groups except PCL/Coll 75% (not significant) displayed a highly significant (*P* < 0.001) relative increase of metabolically active cells compared to the 24 h groups (Figure 2(a)). The count of cells detected after 72 hours is higher compared to the 24 h results throughout all groups (*P* < 0.001) (Figure 2(a)).

Regarding the absolute amount of viable cells after 24 h (Figure 2(b)) there is no statistically significant difference between nanofiber scaffolds containing collagen type I and pure PCL scaffolds, while wells coated with bsGel-TB harbor more viable cells than any nanofiber scaffold group (*P* < 0.001) (Figures 2(b) and 2(c)). Measurements 72 h after cell seeding indicate significantly larger cell populations on PCL/Coll 5% (OD 1.0 ± 0.07) and PCL/Coll 25% (OD 0.94 ± 0.01) compared to pure PCL scaffolds (OD 0.58 ± 0.08) (*P* < 0.001).

### 3.3. Effects of PCL Nanofiber Scaffolds on Cell Proliferation

**Figure 3:**
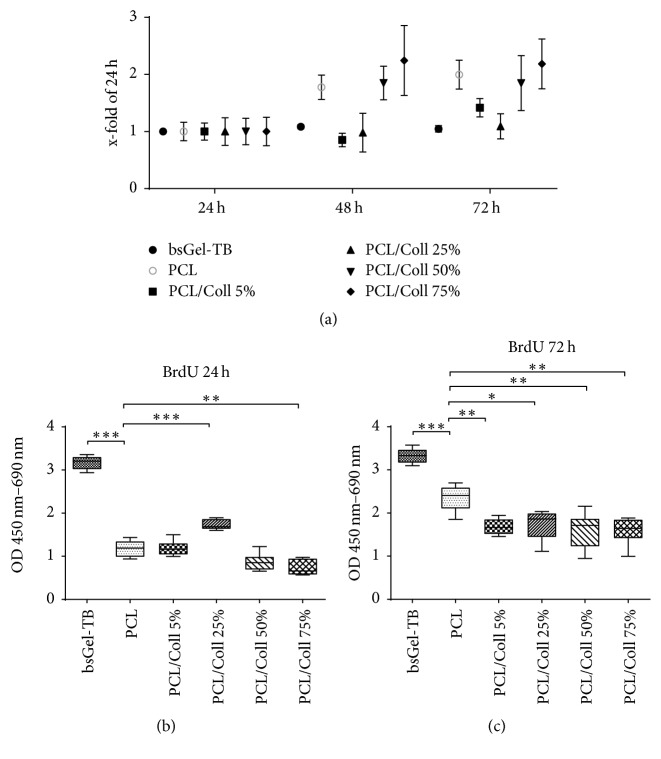
Cell proliferation on electrospun polymer scaffolds and bsGel-TB coated wells. Bromodeoxyuridine (BrdU) integration was detected after 24 h, 48 h, and 72 h and depicted as relative increase compared to 24 h results (a) and as absolute results after 24 h (b) and 72 h (c). Statistically significant differences between the different groups are indicated for ^*∗*^*P* ≤ 0.05, ^*∗∗*^*P* < 0.01, and ^*∗∗∗*^*P* < 0.001.

Regarding the relative increase of proliferation compared to the first 24 hours (Figure 3(a)) cells seeded on bsGel-TB and PCL/Coll 25% showed a quite constant proliferation rate throughout all measurements. The PCL, PCL/Coll 50%, and 75% groups reached their maximum proliferation rate within 48 hours after cell seeding. Cells seeded onto PCL/Coll 5% nanofiber scaffolds showed a slight decrease between 24 and 48 hours and a 56% increase between 48 and 72 h (*P* < 0.001).

Concerning absolute proliferation rate (Figures 3(b) and 3(c)) bsGel-TB appeared superior to nanofiber scaffolds throughout this study (*P* < 0.001). Within the first 24 h (Figure 3(b)) proliferation rate on PCL/Coll 25% (OD 1.74 ± 0.12) scaffolds was higher (*P* < 0.001) than on any other nanofiber scaffolds including pure PCL (OD 1.18 ± 0.19). Proliferation rates on PCL/Coll 75% (OD 0.73 ± 0.18) were significantly lower than on PCL scaffolds.

Between 48 and 72 h after cell seeding pure PCL scaffolds provided higher proliferation rates compared to other nanofiber scaffold groups, while there were no significant differences among blended PCL/Coll groups (Figure 3(c)).

### 3.4. Effects of PCL Nanofiber Scaffolds on Apoptosis

**Figure 4:**
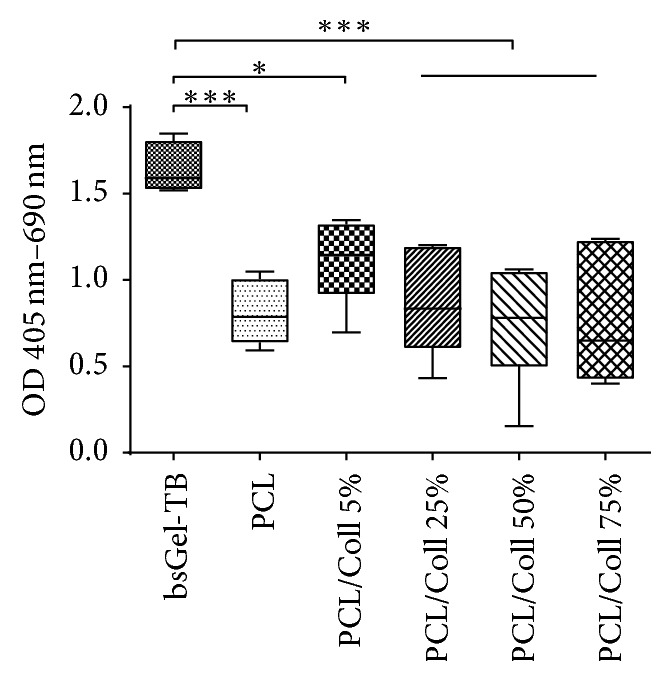
The amount of apoptotic cells was determined via DNA fragmentation ELISA after 3 days. Results show no significant differences among nanofiber scaffold groups, but higher counts of apoptotic cells in gelatine-coated wells. Statistically significant differences between the different groups are indicated for ^*∗*^*P* ≤ 0.05 and ^*∗∗∗*^*P* < 0.001.

The cell death detection ELISA is an assay for quantitative determination of histone-associated DNA fragmentation. The results showed no significant differences between the nanofiber scaffold groups after 72 hours. But the amount of apoptotic cells in gelatine-coated wells (OD 1.64 ± 0.14) was higher than in any other group (mean of all nanofiber scaffold groups: OD 0.85 ± 0.29) (Figure 4)

### 3.5. Effects of PCL Nanofiber Scaffolds on VEGF Production

**Figure 5:**
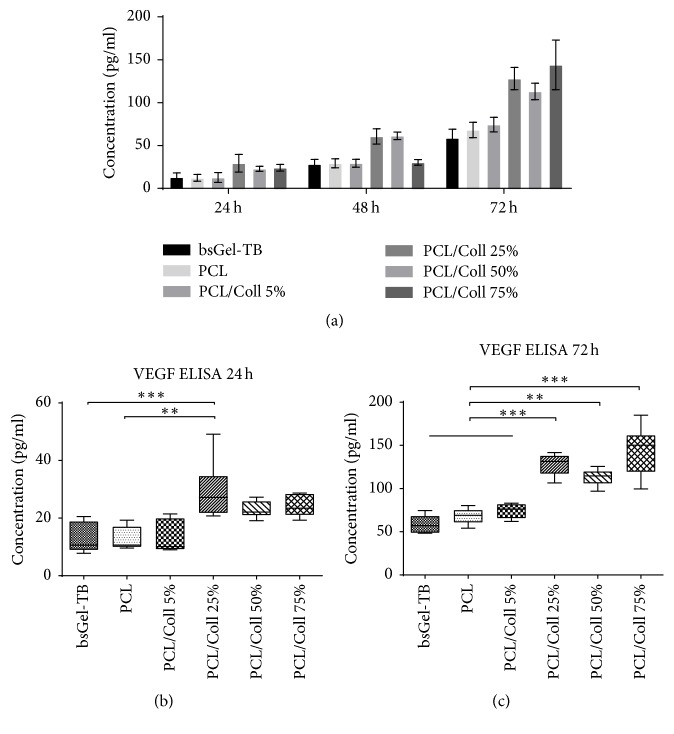
Endothelial cells produce and secrete VEGF. The effect of gelatine-coated wells, PCL, and PCL/Coll nanofiber scaffolds on VEGF secretion of T17b eEPCs was determined from cell culture supernatants after 24, 48, and 72 h. (a) 24 h results show advantages of PCL/Coll 25% scaffolds over pure PCL and bsGel-TB groups (b). After 72 h, measured VEGF concentrations were significantly higher in PCL/Coll 25%, 50%, and 75% than in the pure PCL control group. Statistically significant differences between the different groups are indicated for ^*∗∗*^*P* < 0.01 and ^*∗∗∗*^*P* < 0.001.

The VEGF produced by T17b eEPCs was determined from cell culture supernatants via sandwich ELISA. A constantly increasing concentration of VEGF was detected in all groups (Figure 5(a)).

From day one (24 h) the addition of 25% collagen seems to be advantageous compared to bsGel-TB and pure PCL regarding VEGF production (Figure 5(b)). The 72 h results corroborate this circumstance even further as all groups of nanofiber scaffolds containing ≥25% collagen type I showed significantly (*P* < 0.001) increased concentrations of VEGF compared to bsGel-TB, PCL, and PCL/Coll 5% scaffold groups (Figure 5(c)).

### 3.6. Cell Morphology Study

The morphology of T17b eEPCs was evaluated with both, scanning electron microscopy (SEM) and immunofluorescence microscopy. During the critical point drying process pure PCL scaffolds shrunk to a point to which SEM imaging was no longer possible.

**Figure 6:**
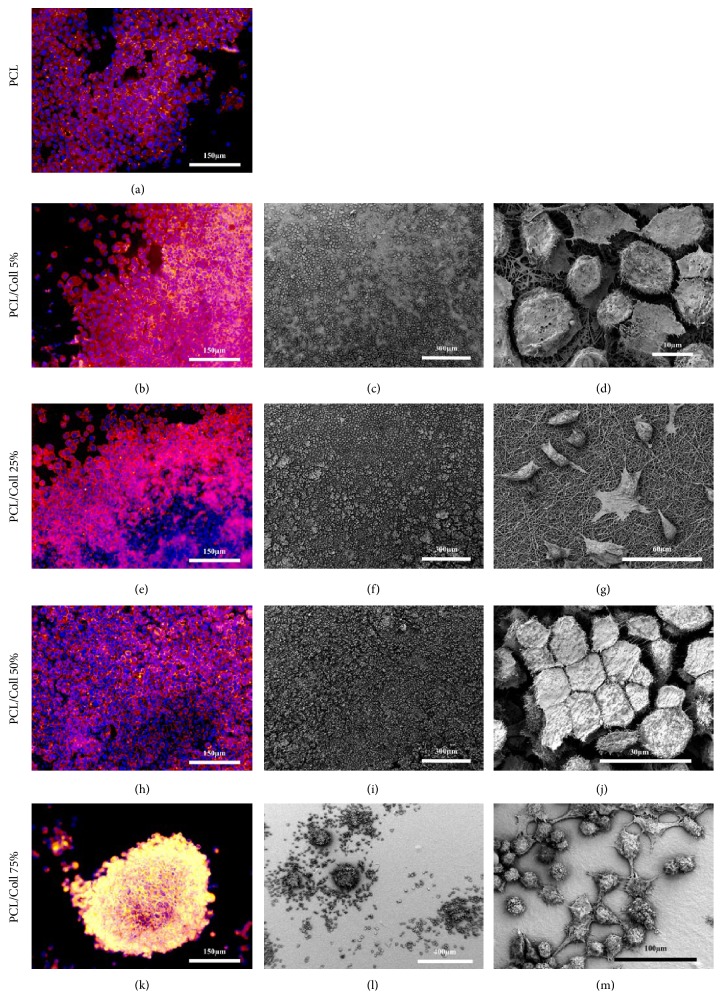
1 × 10^5^ T17b eEPCs were cultivated on nanofiber scaffolds for 3 days and their morphology analyzed via SEM after critical point drying (c, d, f, g, i, j, l, m) reflected light microscopy after immunofluorescent staining (phalloidin + DAPI) (a, b, e, h, k). Pure PCL scaffolds shrunk due to the drying process, making SEM imaging impossible. Cells attach their pseudopodia to nanofibers (here on a PCL/Coll 25% scaffold) (g). PCL/Coll 75% scaffolds lost their ultrastructure. Cells assume a rather round morphology and built large cell clusters (k, l, m). In areas of high cell density “cobblestone” formation can be observed (here found on a PCL/Coll 50% scaffold) (d, j). Cells are torn apart partially, due to the drying process. Nicely spread cell-monolayers and “cobblestone” formations were observed mainly on PCL, PCL/Coll 5%, and 25% scaffolds (a–g, j). Cells seeded on PCL/Coll 50% lost their polarity towards the scaffolds material (h, i).

In general, T17b eEPCs showed a strong adherence to the different nanofiber scaffolds. In most cases, the cells were spread nicely across the surface of the scaffolds. Cells could be observed to attach their pseudopodia to the fibers and even reach into the porous material (Figure 6(g)). Depending on the density of cells, T17b eEPCs assumed different morphologies known to be typical for endothelial cell types. Cells showed a rather flat morphology if fewer cells covered the material (Figure 6(g)). Cells growing in denser formations, built strong connections to neighboring cells, covering not as much material as in flat morphology but instead assuming hexagonal shape and “cobblestone” formation (Figure 6(j)).

Interestingly, the ultrastructure of the material itself was altered during the cell culture process. The PCL/Coll 75% nanofiber scaffolds lost almost their entire ultrastructure within 3 days (Figure 6(m)), while all other scaffolds showed only marginal changes (Figure 6(g)).

Regarding the different nanofiber scaffold groups, the density of eEPCs seemed to be increasing with larger collagen concentrations. Representative images display an increase regarding cell density up to the PCL/Coll 50% group (Figures 6(a)–6(j)). The cells on PCL/Coll 75% formed, in contrast to all other groups, large clusters of cells with apparently no polarity towards the scaffolds material (Figures 6(k) and 6(l)). These clusters showed the highest density of cells found on any scaffold (Figure 6(k))

Besides the cell density, the groups differed, as mentioned already, in the polarity cells had towards the nanofiber scaffolds. While pure PCL (Figure 6(a)) and PCL/Coll 5% (Figures 6(b) and 6(c)) scaffolds were covered with a monolayer of T17b, cells seeded onto PCL/Coll 25% partially lost this feature in areas where the density was extremely high. On PCL/Coll 50% scaffolds cells were stacked onto each other in an apparently chaotic manner, not forming a homogenous surface at all (Figure 6(i)). As most cells found on PCL/Coll 75% were part of a cluster (see above), these cells showed the lowest polarity towards the material.

Furthermore, the “cobblestone” formation of cells was found PCL, PCL/Coll 5%, and 25% scaffolds. Here, the cytoskeleton staining displayed an organized and compact monolayer (Figures 6(a), 6(b), and 6(e)). Populations of T17b on PCL/Coll 50% and 75% scaffolds had rather round cell morphologies (Figures 6(i) and 6(m)).

## 4. Discussion

The integrity of an EC monolayer on the luminal surface of a tissue engineered vascular graft is considered crucial in order to ensure the patency and functionality* in vivo* [27]. To achieve a favorable environment for ECs, many studies attempt to imitate the ECM of native vessels using natural components, supported by biopolymers to meet the required biomechanical features a priori [15, 19].

In this context, PCL meets the requirements for a suitable biopolymer for TEVG production. PCL yields nanofibers with reproducible mechanical and morphological properties. Studies show that tensile stress tests of PCL nanofiber based vascular grafts outweigh those of natural human vessels, thus “facilitating the* in vivo* use as a vascular prosthesis” [18]. Furthermore, PCL TEVGs tested as an abdominal aortic substitute in rats showed good patency rates and marginal intimal hyperplasia [18]. Bearing in mind that collagen I has an impact on endothelial cell physiology such as promoting angiogenesis by regulation of precapillary and multicellular formation [28] or mature endothelial phenotype [29] and promotes cell attachment, we used electrospun nanofibers of collagen I blended PCL.

While others have already investigated collagen-coated PCL nanofiber membranes on endothelial cell physiology [30], our study investigated the influence of different PCL/collagen I ratios on endothelial cell physiology and morphology using scaffolds made of blended electrospun nanofibers. We used the well-established murine endothelial progenitor cell line T17b. Endothelial differentiation was induced with retinoic acid and cAMP as previously verified by an upregulation of endothelial marker genes [26, 31]. In a former study, we seeded T17b eEPCs into a fibrin matrix and proved that T17b EPCs proliferate, form tube-like structures, and secrete VEGF [31]. Since T17b eEPCs do not express MHC I molecules and cannot be detected by natural killer cells, xenogenic transplantation is possible [31]. Furthermore, we demonstrated that T17b eEPCs support the formation of fibrovascular tissue indicated by increased blood vessel quantity and diameter when implanted subcutaneously [32].

The findings of this study comply with the current state of science that PCL supports cell adherence and growth [33]. Our data shows no initial advantage of added collagen type I regarding the amount of viable adherent cells after 24 h (Figure 2(b)), but PCL/Coll 5% and 25% scaffolds host more viable cells compared to pure PCL after 72 h (Figure 2(c)). Interestingly, the proliferation rate 72 h after cell seeding was higher in the PCL group than in any blended group (Figure 3(c)) suggesting that collagen I might have a lesser effect on cell proliferation. On the other hand collagen I can induce the “angiogenic switch” in endothelial cells. Seeding microvascular ECs onto collagen I induces actin polymerization. This mechanism is important for the formation of precapillary cords and specific for collagen. The importance of collagen for angiogenesis can also be deduced from gene expression analysis of tumor endothelium revealing an upregulation of collagen type I and III gene expression. In addition to that, neovascularization has been successfully inhibited in animal models by interfering with the formation of collagen triple helices [34].

Previous work has shown that ECs migrate into a collagen gel, remodel it, and form luminal structures [35, 36], while PCL is known for slow degradation rates [18]. In our study scaffolds with higher collagen I concentrations (75%) appear to be remodeled faster leading to the loss of the scaffold ultrastructure (Figures 6(h), 6(i), 6(k), and 6(l)). This might explain the high-density cell clusters on PCL/Coll 75% scaffolds (Figures 6(k) and 6(l)) and the lower proliferation rate on collagen I blended scaffolds.

The VEGF ELISA provided similarly interesting results. Vascular endothelial growth factor (VEGF) is one key regulator of angiogenesis [37]. It does not only promote vascular growth, but also support EC survival [38]. T17b eEPCs, among other proteins, produce and secrete VEGF [26]. Our data shows an increased production of VEGF in the PCL/Coll 25% group from day one and in all groups containing ≥ 25% collagen I at day 3 (Figure 5) compared to pure PCL and bsGel-TB groups. These findings are concordant with the fact that gene expression is influenced by the surface ECs are attached to [39].

It has been shown that collagen type I can affect apoptosis of adherent cells by downregulation of the proapoptotic Fas [40]. Our data suggests that the surface-integrin interaction on PCL and PCL/Coll nanofiber scaffolds might have an antiapoptotic effect on T17b cells and therefore constitute a better ECM for ECs than gelatine (Figure 4). The addition of collagen I however did not prove to be advantageous over pure PCL, despite higher VEGF concentrations within some PCL/Coll groups. Although VEGF is not the only effector molecule in the regulation of apoptosis, it has been shown to have a measurable antiapoptotic effect [41]. The question still emerges, whether PCL itself has an antiapoptotic effect on ECs. To answer this question, further molecular analysis has to be carried out.

The T17b eEPCs morphology too was largely affected by the composition of the graft material. In contrast to another study investigating collagen-coated electrospun PCL scaffolds [30], endothelial cell monolayer configuration could not be maintained throughout all groups. As the addition of collagen type I increased, the cell configuration changed from an organized monolayer (PCL and PCL/Coll 5%), over higher density multilayered configurations (PCL/Coll 50%), to the formation of large cell clusters (PCL/Coll 75%) (Figure 6).

Also the differences in fiber diameter distribution might have had an effect on EC morphology. Furthermore, the observed period of 72 hours merely depicts a short-term result, leaving long-term developments open for speculation. Nonetheless our study implicates that collagen I has an effect on morphology and configuration of endothelial cells on PCL based nanofiber scaffolds* in vitro*. As mentioned above, this effect is probably associated with the ECs interaction with the scaffolds surface and capability to remodel the material itself.

Admittedly, the cocultivation of T17b eEPCs with smooth muscle cells might reflect the physiological vessel architecture in a more adequate manner. On the other hand, we were able to prove a good cytocompatibility of collagen-blended PCL nanofiber scaffolds. In particular, PCL scaffolds containing 25% collagen seem to be ideal with regard to endothelial cell physiology, morphology, and scaffold integrity: 3 important parameters for vascular tissue engineering. Taking into account the fact that endothelial cell morphology is greatly influenced by shear stress [42, 43] a dynamic cell culture setting with cellularized, electrospun collagen-blended PCL tubes containing 25% collagen might constitute a closer imitation of physiological conditions prior to the* in vivo* application. With regard to* in vivo* implementation, T17b eEPCs are an interesting cell source because they do not express MHC I molecules, making them ideal for testing endothelialized vascular grafts in different animal models [31].

## 5. Conclusion

Electrospun poly-*ε*-caprolactone/collagen I nanofibers constitute a potent extracellular matrix for endothelial cells and therefore are a suitable material for the fabrication of tissue engineered vascular grafts.

Our study underlined that collagen-blended PCL scaffolds feature favorable conditions for endothelial cells regarding the capability to host viable cells, but most importantly they support the functionality of ECs in form of VEGF production and secretion.

But our study also showed adverse effects of collagen type I in PCL nanofiber scaffolds. The higher the collagen : PCL ratio (>50%), the higher the likelihood concerning the loss of an organized EC monolayer or the ultrastructural integrity of the graft itself most likely as a result of accelerated remodeling of the material in contrast to pure PCL.

The overall features of a PCL graft containing 25% collagen type I proved to be ideally balanced regarding the EC physiology and morphology as well as the integrity of the scaffold itself.
